# Central administration of human opiorphin alleviates dextran sodium sulfate-induced colitis in mice through activation of the endogenous opioid system

**DOI:** 10.3389/fphar.2022.904926

**Published:** 2022-09-13

**Authors:** Pan Luo, Xuelin Li, Yuan Gao, Zhengjun Chen, Quanwei Zhang, Zhimin Wang, Xiaozhu Tian

**Affiliations:** ^1^ College of Life Science and Technology, Gansu Agricultural University, Lanzhou, China; ^2^ National Demonstration Center for Experimental Biology Education, School of Life Science, Lanzhou University, Lanzhou, China; ^3^ Gansu Provincial Hospital PET/CT Center, Lanzhou, China

**Keywords:** human opiorphin, inflammatory bowel diseases, ulcerative colitis, endogenous opioid system, intestinal barrier function, enkephalinase inhibitor

## Abstract

The opioid system plays a crucial role in maintaining gastrointestinal homeostasis. Endogenous opioid peptide enkephalins have anti-inflammatory effect and participate in the treatment of inflammatory bowel diseases (IBDs). Here, we investigated the effect of natural enkephalinase inhibitor human opiorphin (HO) on dextran sodium sulfate (DSS)-induced colitis in mice. Our results showed that central administration of HO attenuated DSS-induced colitis, as indicated by the reduction of disease activity index (DAI) scores, macroscopic scores, histological scores, and the myeloperoxidase (MPO) activity. Moreover, HO alleviated DSS-induced inflammation by decreasing inflammatory cytokines TNF-α, IL-6, and IL-1β, and increasing anti-inflammatory cytokine IL-10 in both serum and colon tissues in DSS-treated mice. The potential anti-inflammatory effect of HO at a dose of 40 μg/kg was observed as evidenced by a decrease in nuclear factor κB (NF-κB) p65, toll-like receptor-4 (TLR-4), iNOS, and COX-2. HO also improved intestinal barrier function by enhancing the expression of tight junction proteins. Furthermore, HO treatment significantly inhibited activities of neutral endopeptidase (NEP) and aminopeptidase N (APN), elevated serum enkephalins concentrations, and increased expressions of mu and delta opioid receptors. In addition, pretreatment with opioid receptor antagonist naloxone hydrochloride (NH) compromised the protective effect of HO and aggravated colitis symptoms, as indicated by inhibited anti-inflammatory effects, disrupted intestinal barrier function, and decreased opioid receptor activity. In conclusion, these data indicate that HO protects against DSS-induced colitis by inhibiting TLR4/NF-κB pathway activation and improving intestinal barrier function through activation of the endogenous opioid system. Therefore, targeting the opioid system with peptidase inhibitors intervention would be a novel strategy in the therapy of IBD.

## 1 Introduction

Inflammatory bowel diseases (IBDs), primarily consisting of ulcerative colitis (UC) and Crohn’s disease, are a group of nonspecific inflammatory diseases occurring in the gastrointestinal tract, which are considered to be related to over-expression of pro-inflammatory cytokines and intestinal barrier dysfunction (Hodson, 2016). With an ongoing increase in the incidence and prevalence, IBDs have become a worldwide health problem. The major clinical symptoms of IBDs are abdominal pain, weight loss, diarrhea, and rectal bleeding. It has been reported that multiple factors participate in the development of IBDs, such as genetic, environmental, dietary habits, gut microbiota, and immunologic and inflammatory responses (Chang, 2020). Accumulating evidence demonstrates that excessive activation of the immune system induces the secretion of pro-inflammatory cytokines in the intestinal mucosa and leads to intestinal inflammation (Van Spaendonk et al., 2021). The nuclear factor κB (NF-κB) signaling pathway plays a critical role in regulating the transcription of various pro-inflammatory genes and protein expressions of inflammatory mediators, including tumor necrosis factor (TNF)-α, interleukin (IL)-6, IL-1β, iNOS, and COX-2, which are involved in the progression of intestinal inflammation (Algieri et al., 2014; Song et al., 2019). Although immune impairment and inflammatory response are recognized as pathological causes, the molecular mechanism of IBDs still remains unclear.

Until now, the effective treatments for IBDs are pharmacological therapy and biological therapy. Pharmacological therapy, including anti-inflammatory drugs (non-steroid anti-inflammatory drugs) and immunosuppressant drugs (corticosteroids, mesalamine compounds, azathioprine, methotrexate, anti-TNF-α, and anti-α_4_β_7_ integrin antibodies), and biological therapy are popularly applied in patients with IBDs. However, these drugs usually induce potential side effects, and relapses of IBD symptoms may occur at any time (Singh et al., 2015; Ungaro et al., 2017). Therefore, the mechanisms underlying the etiology and pathogenesis of IBDs, and effective therapeutic strategies need to be further investigated.

Accumulating evidence has reported that the endogenous opioid system participates in maintaining gastrointestinal homeostasis through the regulation of gastrointestinal motility and intestinal integrity. The endogenous opioid system is composed of opioid peptides and three types of opioid receptors, mu opoid receptor (MOR), kappa opioid receptor (KOR), and delta opoid receptor (DOR) (Dietis et al., 2011), which widely distributed in the central and peripheral nervous systems, and even in the surface of different cells in peripheral tissues, such as muscle, immune cells, and gastrointestinal tract (Mosińska et al., 2016). Endogenous opioids like enkephalins, endomorphins, and dynorphins exert anti-inflammatory and anti-tumor effects through modulating the immune system and inflammatory response, and controlling hormone levels in both human beings and animal models of inflammatory bowel diseases (Owczarek et al., 2011; Wilenska et al., 2018; Tuo et al., 2020). However, these peptides are rapidly metabolized by endogenous peptidases, which are well distributed in central and peripheral tissues, and participate in the regulation of pathophysiological processes in the gastrointestinal tract (Gabrilovac et al., 2004). For example, enkephalin-degrading enzymes, such as neutral endopeptidase (NEP, CD10, EC 3.4.21.11), aminopeptidase N (APN, CD13, EC 3.4.11.2), dipeptidyl peptidase III (DPP Ⅲ, EC 3.4.14.4), and angiotensin-converting enzyme (ACE, EC 3.4.15.1), are involved in the degradation and inactivation of enkephalins in human plasma, and significantly block anti-inflammatory actions of these peptides (Marini et al., 1990; Janecka et al., 2008; Sałaga et al., 2013). Given the anti-inflammatory effect of endogenous opioids, peptidase inhibitors could play important roles in controlling inflammatory diseases. Therefore, owing to the physiological importance and the critical role of NEP and APN in enkephalins inactivation, enkephalinase inhibitors have been studied for several years, which may be a promising therapeutic strategy in the treatment of IBDs. Enkephalinase inhibitor acetorphan has been proven to exhibit naloxone-reversible anti-diarrheal activity in rodents *via* the protection of endogenous enkephalins (Marҫais-Collado et al., 1987). Similarly, thiorphan, a specific NEP inhibitor, is currently used clinically as a potent anti-diarrhea drug and alters intestinal motility in fed rats mediated by an endogenous opioid pathway (Sałaga et al., 2013). Recently, natural enkephalinase inhibitors of NEP and APN, human opiorphin (HO), rat sialorphin, and bovine spinorphin, have been identified and extensively studied. To date, spinorphin is examined to have potential anti-inflammatory activity by inhibiting APN in the mouse air-pouch assay (Yamamoto et al., 1998). Sialorphin and its analog are demonstrated to exhibit anti-inflammatory effect on 2,4,6-trinitrobenzene sulphonic acid (TNBS)-induced colitis through MOR and KOR (Sałaga et al., 2017). Therefore, we speculate that HO may have anti-inflammatory effects.

HO, isolated from human saliva, is identified as a natural enkephalinase inhibitor of NEP and APN. Sufficient evidence suggests that HO protects enkephalins from enzymolysis both *in vivo* and *in vitro* (Wisner et al., 2006; Tian et al., 2009). HO has been implicated in several biological actions in mice, including analgesic activity, penile erection, and cardiovascular functions (Tong et al., 2008; Tian et al., 2009; Tian et al., 2015). Our previous study suggests that HO enhances colonic contraction by activating MOR and DOR *in vitro*, which is attributable to the inhibition of enkephalins degradation (Tian et al., 2009). Considering the multiple activities of HO and the anti-inflammatory effect of endogenous enkephalins, the hypothesis was that HO may be involved in maintaining gastrointestinal homeostasis and modulating immune inflammatory response. Therefore, in the present study, we evaluated the effect of the central administration of HO on dextran sulfate sodium (DSS)-induced colitis in mice and confirmed its anti-inflammatory activity associated with opioid receptor-dependent enkephalinergic pathways. Moreover, we also investigated the effect of HO on intestinal motility and barrier function, and further verified that HO is involved in regulating intestinal homeostasis. Our findings would provide a theoretical basis for future clinical research in the treatment of IBD.

## 2 Materials and methods

### 2.1 Drugs

HO was synthesized by the solid-phase peptide synthesis method, based on our previous study (Tian et al., 2009). All Fmoc-protected amino acids were purchased from GL Biochem Co., Ltd. (Shanghai, China). The separation, purity, and preparation of the peptide were performed by high-performance liquid chromatography. The purity of HO exceeded 98.5%. DSS (MW: 36,000–50000) was purchased from Yeasen Biotech Co., Ltd. (Shanghai, China), 5-aminosalicylic acid (5-ASA) was purchased from Sigma-Aldrich (Germany), and naloxonazine dihydrochloride (NH) and pentobarbital sodium were obtained from Merk (Darmstadt, Germany). All reagents were dissolved in phosphate-buffered saline (PBS, pH = 7.2; NaCl 8 g/L, KCl 0.2 g/L, Na_2_HPO_4_ 1.44 g/L, KH_2_PO_4_ 0.24 g/L).

### 2.2 Experimental animals

Ten-week-old adult male C57BL/6 mice (25 ± 2 g) were obtained from the Animal Center of Lanzhou University (Lanzhou, China, SCXK (GAN) 2018-0002). All mice were housed in a specific pathogen-free separate room at 22 ± 2 °C with a 12 h light–dark cycle and 40–60% relative humidity, and adapted for 5 days before the experiments (Nagata et al., 2021; Wu et al., 2021; Chen et al., 2022). Standard food and water were freely available in this period. All animal protocols were performed according to Lanzhou University’s Institutional Animal Care and Use Committee guidelines (Lanzhou, China). Every effort was made to minimize animals’ suffering and reduce the number of mice used in the experiments.

### 2.3 Experimental protocols

#### 2.3.1 Surgery

Eighty-eight C57BL/6 mice were anesthetized by intraperitoneal injection of pentobarbital sodium (80 mg/kg of body weight) and fixed to a stereotaxic apparatus (DW-2000, Chengdu Taimeng Software Co. Ltd., China), and then each mouse was implanted with a guide cannula in the lateral ventricle for intracerebroventricular (i.c.v.) administration of HO. Based on a previous study (Sakamoto and Endo, 2008), the site of catheter implantation was 1.5 mm from the middle, 1 mm from the bregma, and 3 mm from the surface of the skull. The guide cannula was fixed to the skull with dental cement. The tip of the internal cannula (C315IS-5, 33 gauge) for microinjection was inserted 1 mm below the tip of the guide cannula. After surgery, mice were allowed to recover for 5 days before treatment. All mice were randomly divided into 11 groups (*n* = 8 per group), and each mouse was kept in a separate cage.

#### 2.3.2 Dextran sulfate sodium-induced colitis and treatment protocols

**FIGURE 1:**
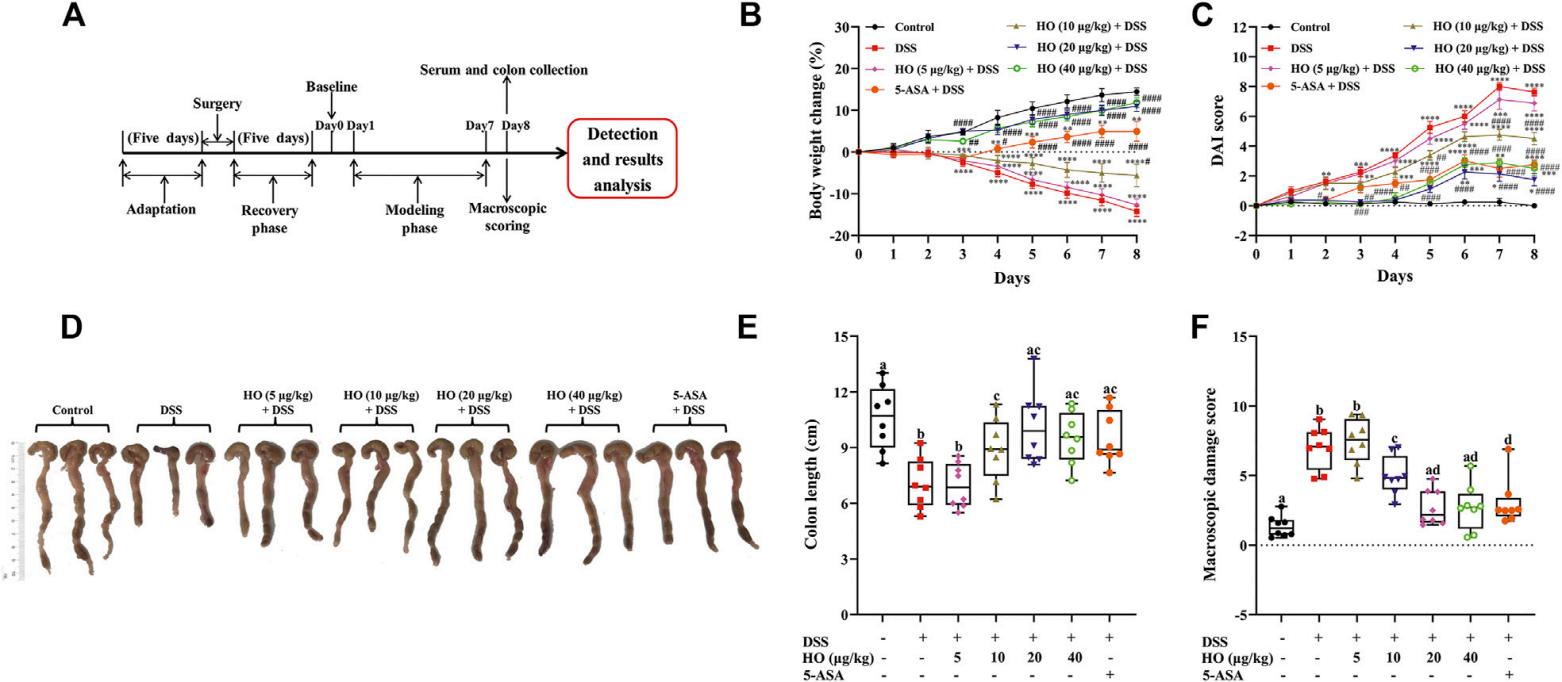
Effects of human opiorphin treatment on macroscopic colonic damage in DSS-induced colitis in mice. **(A)** Experimental design in this experiment. **(B)** Body weight change. The body weight change was calculated as the percentage of weight gain or loss in relation to baseline weight for each mouse. **(C)** DAI score. **(D)** Macroscopic images of colons. **(E)** Colon length. **(F)** Macroscopic damage score. The data are expressed as mean ± S.E.M. (*n* = 8). ^*^ *p* < 0.01, ^**^ *p* < 0.01, ^***^ *p* < 0.005, ^****^ *p* < 0.0001 *vs.* control group, and ^#^ *p* < 0.05, ^##^ *p* < 0.01, ^###^ *p* < 0.005, ^####^ *p* < 0.0001 *vs.* DSS group, one-way ANOVA with Tukey’s multiple comparisons test; bars with different letters represent significant differences between groups by Fish’s LSD test (*p* < 0.05).

DSS-induced colitis in mice was performed by the administration of 2.5% DSS (w/v) in drinking water for 7 days. The DSS solution was freshly prepared every other day; 5-ASA (100 mg/kg body weight) was used as a positive control and was orally gavaged once daily for 8 days. HO and PBS were injected into the lateral ventricle at a volume of 2 μl at a constant rate of 2 μl/min with a microsyringe. In order to ensure drug absorption, after each injection, the injection cannula was kept *in situ* for 1 min; 7 groups of mice were chosen to evaluate the anti-inflammatory effects of HO on DSS-induced colitis in mice: 1) the control group (i.c.v. administration of PBS), 2) DSS group (mice treated with DSS and i.c.v. administration of PBS), 3) HO (5 μg/kg) + DSS group (mice treated with DSS and i.c.v. administration of HO at a dose of 5 μg/kg body weight), 4) HO (10 μg/kg) + DSS group (mice treated with DSS and i.c.v. administration of HO at a dose of 10 μg/kg), 5) HO (20 μg/kg) + DSS group (mice treated with DSS and i.c.v. administration of HO at a dose of 20 μg/kg), 6) HO (40 μg/kg) + DSS group (mice treated with DSS and i.c.v. administration of HO at a dose of 40 μg/kg), and 7) 5-ASA + DSS group (mice treated with DSS and orally administered 5-ASA). HO, PBS, and 5-ASA treatment for the first time are carried out 30 min before DSS (Sałaga et al., 2017). HO and PBS were i.c.v. administrated twice daily with an interval of 2 hours for 8 days. In addition, the mice in the other four groups were used to investigate the mechanisms underlying the protective effect of HO on colitis. As described in our previous study (Tian et al., 2009), opioid receptor antagonist NH (2 mg/kg body weight) was subcutaneously injected 10 min before i.c.v. administration of HO (40 μg/kg) twice daily. NH treatment alone did not influence the observed parameters compared to the control group. The experimental design in the study is shown in Figure 1A.

The body weight of each mouse was recorded from 0 to 8 days. The 0 day body weight acted as the baseline, which was used to calculate the percentage of body weight change of each mouse during the test. Body weight change, stool consistency, and the presence or absence of blood in feces were monitored daily to evaluate the disease activity index (DAI). After 7 days of treatment, gut motility and permeability assays were performed. The serum of each mouse was collected 1 hour after the last treatment of PBS or HO, and stored at −80°C for further investigations. All mice were killed by cervical dislocation. The colon was collected, and its length, weight, and thickness were measured. The dots of ulceration in the entire colon were also counted. Then, the colon was cut into small pieces and fixed with 4% paraformaldehyde for histological analysis. The remaining colon tissues were immediately exposed to liquid nitrogen and stored at −80°C for other analysis.

### 2.4 Evaluation of colitis severity

#### 2.4.1 Macroscopic colonic damage

DAI, including relative body weight loss, stool consistency, and fecal blood, is widely used to evaluate the development of clinical symptoms of colitis. Therefore, these parameters were monitored daily. Fecal occult blood was detected by a benzidine probe and visual pellet bleeding or hemorrhage. DAI was represented as the average of scores for body weight loss, stool consistency, and fecal blood, the scoring criteria for which are described in Supplementary Table S1. Moreover, the macroscopic damage score of each mouse was calculated based on dots of ulceration in the colon, colon shorting and thickness, diarrhea, fecal blood, and hemorrhage. The criteria for scoring macroscopic colonic damage are shown in Supplementary Table S2.

#### 2.4.2 Histological colonic damage

After 7 days of treatment, the severity of colitis was evaluated using the histopathological study of colonic tissue. According to the previously described Swiss-rolling technique (Bai et al., 2016), the entire colon was excised and fixed in 4% formaldehyde as a Swiss roll, and then embedded in paraffin. Tissue sections of 5 μm thickness were stained with hematoxylin and eosin (Beijing Solarbio Science and Technology, Beijing, China). The slides were then studied using a light microscope for observation and photography. The histological scoring of the colon was evaluated according to the method described previously (Sałaga et al., 2017). For scoring colonic histological damage, each colonic tissue specimen was graded based on goblet cell depletion, crypt abscesses, the extent of muscle thickening, destruction of the mucosal layer, and degree of inflammatory cell infiltration. The criteria for scoring histological colonic damage are shown in Supplementary Table S3.

### 2.5 Immunohistochemical analysis

To study the intestinal mucosal barrier, mucin-2 in colonic tissues of each mouse was detected by immunohistochemistry assays. First, the entire colon was excised and fixed in 4% formaldehyde as a Swiss roll and embedded in paraffin, and then cut to prepare 5-µm-thick paraffin sections. Tissue paraffin sections were deparaffinized and incubated with 3% hydrogen peroxide for 20 min. Then, sections were incubated at 4°C overnight with primary antibody rabbit anti-mucin-2 (A01212, 1:100, Boster Biological Technology co., Ltd., Wuhan, China). Staining was performed using a two-step immunohistochemical staining kit (Boster Biological Technology co., Ltd., Wuhan, China) according to the manufacturer’s instructions. Sections were stained with a diaminobenzidine kit (Beijing zhongshanjinqiao Biotechnology Co., Ltd., Beijing, China), and then counterstained with hematoxylin. At last, the stained tissue slides were visualized under a light microscope and examined to obtain images. The semi-quantitative analysis of mucin-2 was performed by measuring the integrated optical density (IOD) value by Image-Pro Plus 6.0 (Media Cybernetics, Inc., Bethesda, MD, United States).

### 2.6 Immunofluorescence analysis

Apoptosis was evaluated by analyzing the presence of active caspase 3 (ACasp-3). ACasp-3 presence was determined by using a rabbit antibody against cleaved caspase-3 (9664T, 1:400, Cell Signaling Technology, Danvers, United States). Immunofluorescence staining was performed according to the manufacturer’s protocols. In brief, paraffin-embedded tissue was sectioned, treated with xylene, and rehydrated, and then colonic sections were incubated with primary antibody at 4°C overnight. AlexaFluor488-labeled secondary antibodies (Jackson ImmunoResearch, United States) were added for 1 h at room temperature. Representative images were taken using a microscope (Olympus, Japan).

### 2.7 Myeloperoxidase activity analysis

Myeloperoxidase (MPO), a specific biomarker of neutrophil infiltration into the inflamed colonic mucosa, was measured to evaluate the severity of the colonic inflammatory response; 200 mg colonic sample of each mouse was prepared by homogenization in PBS and centrifugation was performed at 15,000 rpm for 10 min at 4°C. The supernatant was collected and used to analyze the MPO activity using commercial kits purchased from Nanjing Jiancheng Biology Research Institute (Nanjing, China) according to the manufacturer’s protocols. The results were determined using a spectrophotometer at 460 nm and quantified as nU/mg tissue.

### 2.8 Enzyme-linked immunosorbent assay

Serum enkephalins were measured using mouse enkephalins enzyme-linked immunosorbent assay (ELISA) kits (Jingkang Bioengineering Co., Ltd. Shanghai, China) according to the instructions; 200 mg colonic sample of each mouse was weighed and homogenized with PBS, and then centrifuged at 15,000 rpm for 10 min at 4°C to get the supernatant. The levels of TNF-α, IL-1β, IL-6, and IL-10, and activities of NEP and APN in colonic supernatant and serum were examined utilizing ELISA kits (Jianglai Biotechnology Co., Ltd., Shanghai, China) based on the manufacturer’s instructions.

### 2.9 Gene expression assay

Total RNA samples from the colon of each mouse were extracted using the TRIzol reagent (ShineGene Molecular Biotech. Inc., Shanghai, China). Real-time quantitative polymerase chain reaction (RT-qPCR) was implemented using SYBR Green Master Mix on instrument FTC3000 (Canada). Complementary DNA synthesized from total RNA was initially activated at 94°C for 4 min, 35 cycles at 72°C for 30 s, consisting of a 20 s denaturation at 94°C, and annealing temperature at 60°C for 25 s. The sequences of the forward and reverse primers used in the study have been listed in Table 1. Analyses of all samples were run in triplicate. The relative gene expression of *TNF-α*, *IL-1β*, *IL-6*, *IL-10*, *claudin-1*, *occludin*, *E-cadherin*, *zonula occludens-1* (*ZO-1*), *MOR1*, *DOR1*, *KOR1*, *NEP*, and *APN* were calculated by the 2^−ΔΔCt^ method. Mouse *β-actin* was used as a control to normalize the transcript levels of each biomarker. Relative levels of the mRNA expression were presented as fold change with setting the value of the control group mice as 1.

**TABLE 1:** Primer sequences used in the study.

Gene	Forward primer (5′ to 3′)	Reverse primer (5′ to 3′)
*TNF-α*	CCC​TCC​AGA​AAA​GAC​ACC​ATG	CAC​CCC​GAA​GTT​CAG​TAG​ACA​G
*IL-1β*	GCT​TCA​GGC​AGG​CAG​TAT​CA	TGC​AGT​TGT​CTA​ATG​GGA​ACG
*IL-6*	GTT​GCC​TTC​TTG​GGA​CTG​ATG	CTC​ATT​TCC​ACG​ATT​TCC​CAG
*ZO-1*	GAT​GAG​CGG​GCT​ACC​TTA​CTG	CCTGTCATGCGAGCGACC
*Claudin- 1*	TCA​GGT​CTG​GCG​ACA​TTA​GTG	CAGAAGGCAGAGGGAGGC
*Occludin*	TCC​TGG​AGG​TAC​TGG​TCT​CTA​CG	CCA​TCT​TTC​TTC​GGG​TTT​TCA​C
*E-cadherin*	AAC​AGG​GAC​AAA​GAA​ACA​AAG​G	TGA​TGA​CAC​GGC​ATG​AGA​ATA​G
*MOR1*	TCT​TCA​CCC​TCT​GCA​CCA​TG	TCT​ATG​GAC​CCC​TGC​CTG​TA
*DOR1*	GCT​CGT​CAT​GTT​TGG​CAT​C	AAG​TAC​TTG​GCG​CTC​TGG​AA
*KOR1*	TCC​TTG​GAG​GCA​CCA​AAG​TCA​GGG	TGG​TGA​TGC​GGC​GGA​GAT​TTC​G
*NEP*	CTC​TCT​GTC​TTG​TCT​TGC​TC	GAC​GTT​GCG​TTT​CAA​CCA​GC
*APN*	AGC​TCC​ACA​CAC​CGT​TCC​TG	GCC​CTT​GGC​CAT​GGT​GGT​GAT​GGT​G
*Mouse β-actin*	GAG​ACC​TTC​AAC​ACC​CCA​GC	ATG​TCA​CGC​ACG​ATT​TCC​C

*TNF*, tumor necrosis factor; *IL*, interleukin; *ZO-1*, zonula occludens-1; *MOR*, mu opoid receptor; *DOR*, dalta opioid receptor; *KOR*, kappa opioid receptor; *NEP*, neutral endopeptidase; *APN*, aminopeptidase N.

### 2.10 Western blot analysis

Total protein extraction from colon tissue samples of each mouse was performed using radioimmunoprecipitation assay buffer (50 mmol Tris, pH 7.4, 150 mmol NaCl, 1% Nonidet P-40, 0.5% sodium deoxycholate, and protease inhibitors Phenylmethylsulfonyl fluoride 1 mmol/L) and centrifuged at 12,000 rpm for 20 min. The supernatant was collected and the total protein concentrations were examined using a bicinchoninic acid kit; 20 μg proteins were loaded onto 5–12% SDS-PAGE gels. After electrophoresis, protein bands were transferred to 0.45 μm nitrocellular membranes (Millipore, United States). The membranes were blocked in 5% BSA-TBST for 60 min at room temperature, and then probed with rabbit antibody against iNOS (13120S, 1:1000, Cell Signaling Technology, Danvers, United States), rabbit antibody against COX2 (12282S, 1:1000, Cell Signaling Technology, Danvers, United States), rabbit antibody against TLR-4 (19811-1-AP, 1:2000, Proteintech Group, Inc., Chicago, United States), mouse antibody against NF-κB P65 (ER-180223, 1:2000, Erwantech, Shanghai, China), anti-MOR1 rabbit polyclonal antibody (PA5-26138, 1:1000, Thermo Fisher Scientific Inc., Waltham, MA, United States), rabbit antibody against cleaved ACaps-3 (CST-9664T, 1:2000, Danvers, United States), anti-DOR1 rabbit polyclonal antibody (PA1-4560, 1:1000, Thermo Fisher Scientific Inc., Waltham, MA, United States), and anti-KOR1 rabbit polyclonal antibody (PA5-40216, 1:1000, Thermo Fisher Scientific Inc., Waltham, MA, United States) overnight at 4°C. The membranes were washed with TBST buffer (Erwantech, Shanghai, China) and incubated with horseradish peroxidase-conjugated secondary antibody goat anti-rabbit IgG (1:5000, Erwantech, Shanghai, China). After washing with TBST buffer, the membranes were treated with the enhanced chemiluminescence (Millipore, United States) Western blotting reagent. Rabbit anti-β-actin antibody (BM5422, 1:2000, Boster, Wuhan, China) was used as internal inference and analysis was performed under the same conditions as the target proteins. At last, the density of each protein band was detected and quantified using Image-Pro Plus 6.0, and the ratio of each target protein to β-actin was calculated.

### 2.11 Intestinal permeability assay

Intestinal permeability was assessed using 4000 Da fluorescent-dextran-FITC (Dx-FITC; Sigma-Aldrich, United States). As described in our previous study (Tian et al., 2020), after 5 h fasting, mice were orally gavaged with Dx-FITC (100 mg/ml, 200 µl); 200 µl blood of each mouse was collected 4 h later and centrifuged at 4°C at 5,000 rpm for 10 min. The serum was collected and immediately determined. Intestinal permeability was examined by measuring serum Dx-FITC using a fluorescence spectrophotometer (excitation 485 nm and emission 535 nm).

### 2.12 Statistical analysis

All data are represented as the mean ± S.E.M. The statistical analyses between groups were performed by one-way analysis of variance (ANOVA) with Tukey’s multiple comparisons test or Fisher’s least significant difference (LSD) test using GraphPad Prism version 8.2.1 (GraphPad Software, United States). *p* < 0.05 indicates statistical significance. Principal component analysis (PCA) was performed using R software (version 3.1.0), in order to study the scattering of the protective effect of HO on DSS-induced colitis in mice.

## 3 Results

### 3.1 Human opiorphin prevents dextran sodium sulfate-induced colitis in mice

To examine the protective effect of HO on DSS-induced colitis in mice, 56 male mice were randomly equally divided into seven groups after lateral ventricle catheterization. The control group mice were given drinking water freely and PBS. The DSS group mice were treated with DSS in drinking water and PBS. The 5-ASA + DSS group mice were orally administrated with 5-ASA accompanied with DSS in drinking water. Another four groups of mice were treated with HO at different doses (5, 10, 20, and 40 μg/kg, respectively) accompanied with DSS. The DSS-induced mice colitis model was performed by administration of 2.5% DSS (w/v) in drinking water for 7 days. PBS and HO were i.c.v. administrated twice daily at 2-h intervals for 8 days. After 7 days of treatment, DSS exposure induced a significant decrease in body weight from day two onward followed by a remarkable increase in DAI scores. In contrast, DAI of DSS-exposed mice was significantly improved by HO supplementary at the dose of 10, 20, and 40 μg/kg through attenuating stool consistency and fecal blood (Figures 1B,C). Moreover, compared to the DSS group mice, treatment of HO at the dose of 20 and 40 μg/kg recovered the colon length and colon thickness, reduced the dots of ulceration in DSS-exposed mice, and thereby significantly attenuated the colonic macroscopic damage induced by DSS (Figures 1D–F and Supplementary Table S4).

**FIGURE 2:**
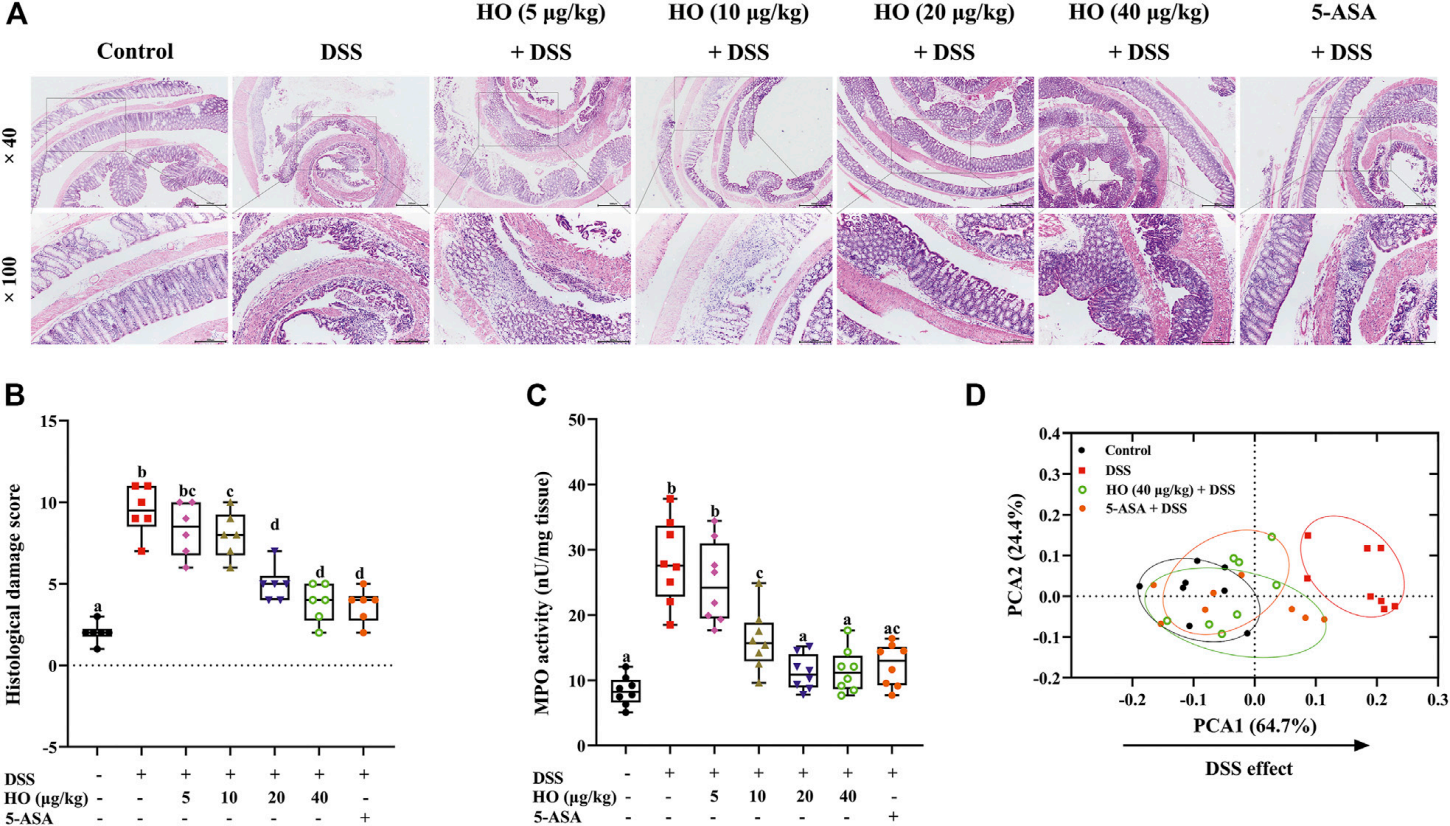
HO treatment prevents DSS-induced colitis in mice. **(A)** Photomicrographs of mice colonic sections stained by hematoxylin and eosin. **(B)** Histological damage score. **(C)** MPO activity. **(D)** PCA for colitis parameters of four different treatment groups. The data are expressed as mean ± S.E.M. (*n* = 6–8). Bars with different letters represent significant differences between groups by Fish’s LSD test (*p* < 0.05).

In addition, HO at the dose of 40 μg/kg prevented DSS-induced histological colonic damage (*p* < 0.0001), as shown in histological sections of the colon on the basis of goblet cell depletion, crypt abscesses, destruction of the mucosal layer, and inflammatory cellular infiltration (Figures 2A,B and Table 2). Furthermore, treatment with HO markedly reduced the colonic MPO activity (*p* < 0.0001) in DSS-exposed mice compared with the DSS group (Figure 2C).

**TABLE 2:** Microscopic colonic damage score in each group mice.

Group	Goblet cell depletion	Crypt abscess	Extent of muscle thickening	Destruction of the mucosal layer	Cellular infiltration
Control	0.333 ± 0.211^a^	0.333 ± 0.211^a^	0.333 ± 0.211^a^	0.667 ± 0.211^a^	0333 ± 0.211^a^
DSS	1.000 ± 0.000^bc^	1.000 ± 0.000^b^	2.500 ± 0.224^b^	2.500 ± 0.224^b^	2.500 ± 0.342^b^
HO (5 μg/kg) + DSS	1.000 ± 0.000^b^	1.000 ± 0.000^b^	1.833 ± 0.307^bc^	2.333 ± 0.211^b^	2.167 ± 0.401^b^
HO (10 μg/kg) + DSS	1.000 ± 0.000^b^	1.000 ± 0.000^b^	1.667 ± 0.211^bd^	2.500 ± 0.224^b^	1.833 ± 0.401^bc^
HO (20 μg/kg) + DSS	0.667 ± 0.211^ac^	0.833 ± 0.167^b^	1.167 ± 0.167^cd^	1.333 ± 0.211^c^	1.000 ± 0.258^ac^
HO (40 μg/kg) + DSS	0.500 ± 0.224^a^	0.667 ± 0.211^ab^	1.167 ± 0.307^cd^	0.667 ± 0.211^a^	0.833 ± 0.167^a^
5-ASA + DSS	0.333 ± 0.211^a^	1.000 ± 0.000^b^	1.000 ± 0.365^ad^	0.667 ± 0.211^a^	0.667 ± 0.211^a^

Microscopic scoring is assessed based on the criteria for histological damage parameters. Values are expressed as mean ± S.E.M. (*n* = 6). Different letters in the same column represent significant differences between groups by Fish’s LSD test (*p* < 0.05). HO, human opiorphin; DSS, dextran sulfate sodium.

Overall, based on colonic damage parameters, including macroscopic scores, histological scores, and MPO, PCA was performed. The results of PCA showed that DSS separated DSS group mice clearly from the control group along PCA1 (64.7%), while HO treatment shifted the HO + DSS groups at the doses of 40 μg/kg toward the control group (Figure 2D). Moreover, HO (40 μg/kg) had the same effect as positive drug 5-ASA (100 mg/kg). These data indicate that HO effectively prevents DSS-treated mice from colitis.

### 3.2 Anti-inflammatory effects of human opiorphin on dextran sodium sulfate-induced colitis in mice

**FIGURE 3:**
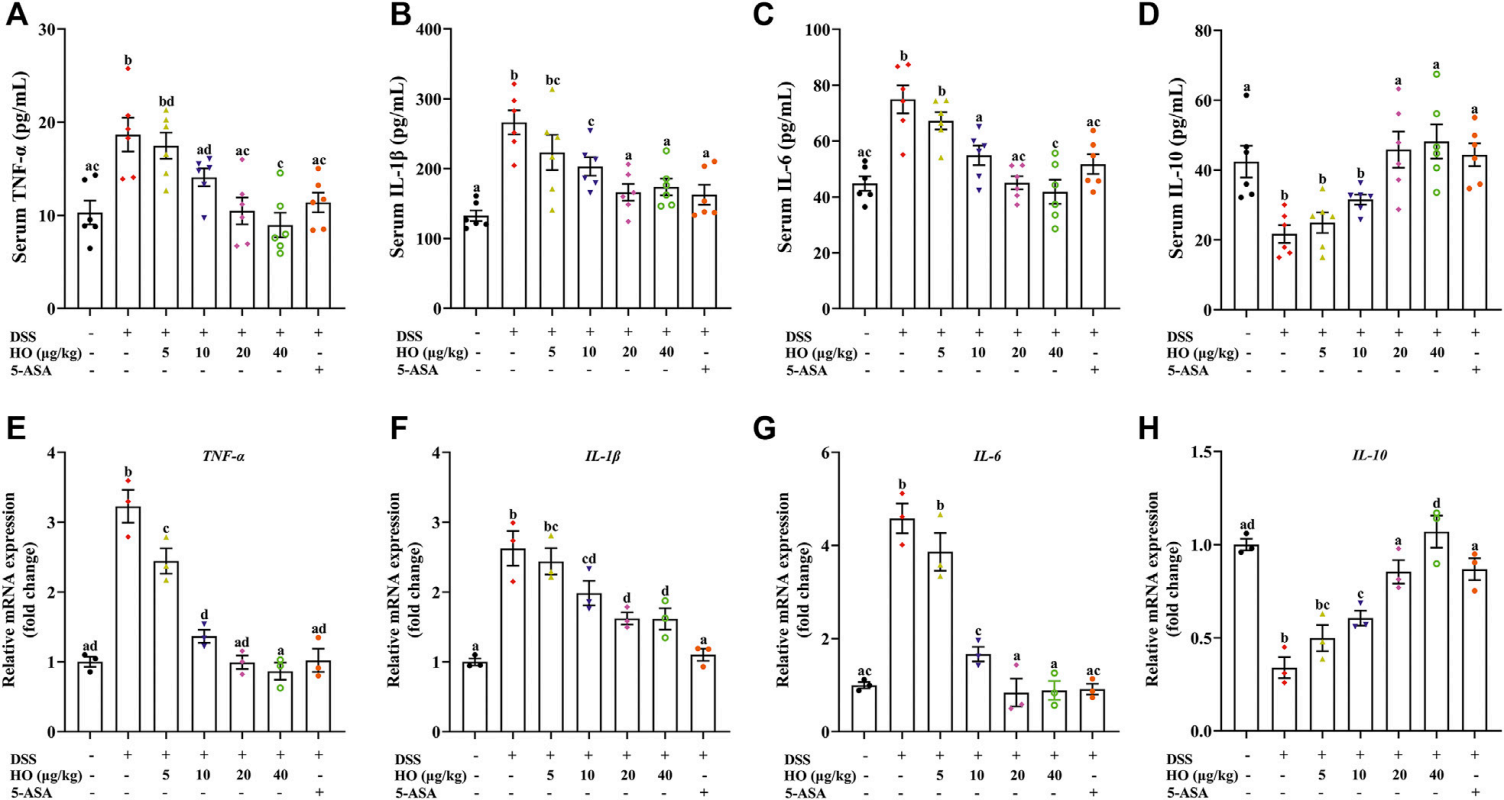
Effect of HO on inflammatory factors in the serum and colon in DSS-exposed mice. **(A)** Serum TNF-α level. **(B)** Serum IL-1β level. **(C)** Serum IL-6 level. **(D)** Serum IL-10 level. Relative gene expressions of TNF-α **(E)**, IL-1β **(F)**, IL-6 **(G)**, and IL-10 **(H)** in the colon. The levels of serum TNF-α, IL-1β, IL-6, and IL-10 were detected by ELISA. Gene expression levels of these cytokines in the colon were analyzed by RT-qPCR. The data are expressed as mean ± S.E.M. (*n* = 3–6). Bars with different letters represent significant differences between groups by Fish’s LSD test (*p* < 0.05).

As shown in Figure 3, compared to the control group, DSS exposure significantly increased the concentrations of inflammatory cytokines including TNF-α, IL-6, and IL-1β (*p* < 0.0001), and decreased the level of the anti-inflammatory cytokine IL-10 (*p* = 0.0004) in the serum of DSS group mice. However, HO administration at doses of 10, 20, and 40 μg/kg markedly inhibited the changes of these cytokines induced by DSS, and at doses of 20 and 40 μg/kg recovered the levels of TNF-α, IL-6, and IL-10 to that of the control group mice (Figures 3A–D). Moreover, HO alleviated inflammatory response in DSS-exposed mice, as indicated by the significant reduction of gene expressions of pro-inflammatory cytokines TNF-α, IL-6, and IL-1β in the colon (Figures 3E–G). Treatment with HO also markedly increased colonic IL-10 mRNA levels (Figure 3H). Therefore, these results indicate that HO has anti-inflammatory properties and attenuates DSS-induced inflammation in the colon of mice with colitis. Importantly, HO at the dose of 40 μg/kg significantly increased the gene expression of IL-10 in the colonic tissue compared to the HO (20 μg/kg) + DSS group and might display potent anti-inflammatory activities. Therefore, we chose the 40 μg/kg dose in our study to evaluate the mechanisms of action in the following study.

### 3.3. Human opiorphin alleviates dextran sodium sulfate-induced colitis in mice through the NF-κB signaling pathway by activating the opioid pathway

**FIGURE 4:**
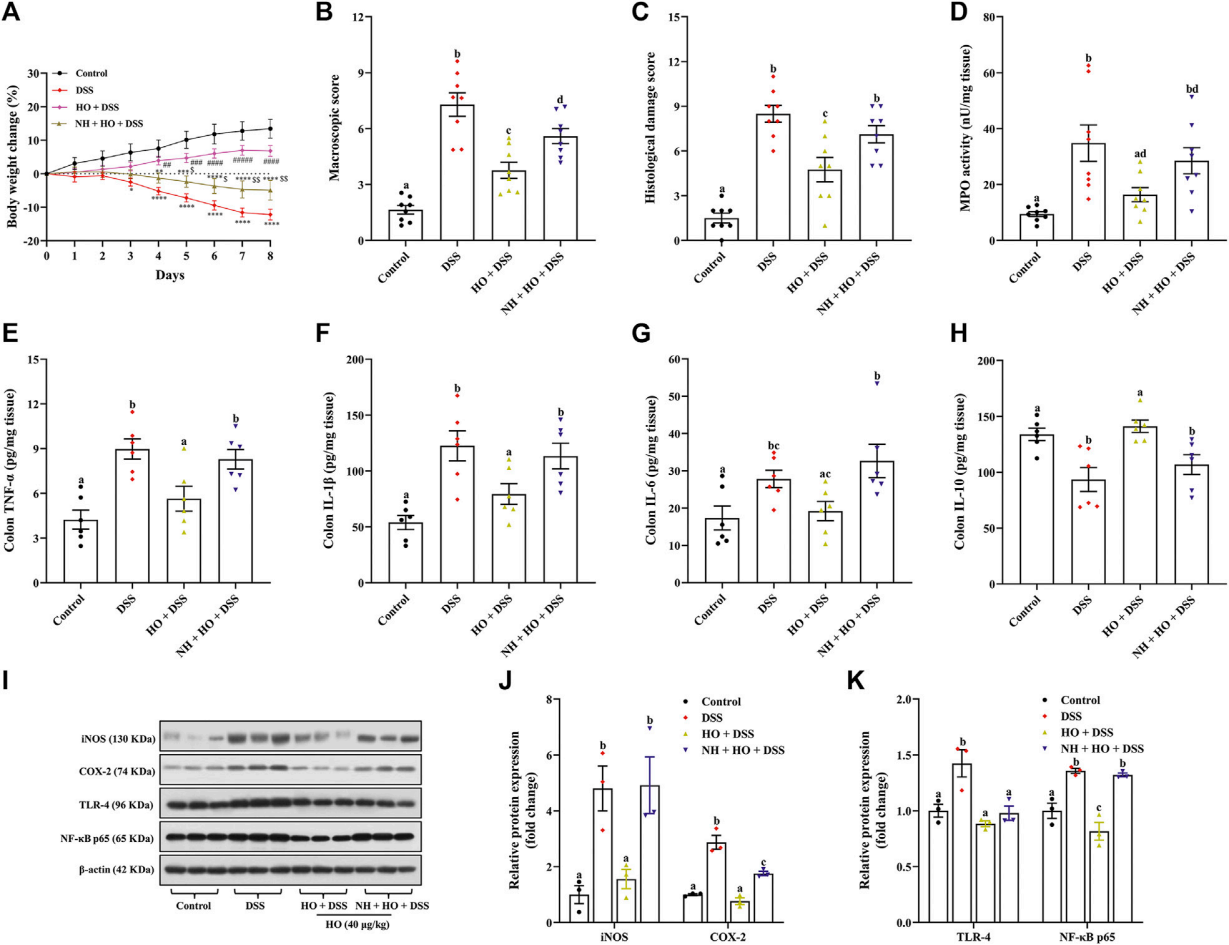
HO exhibits anti-inflammatory effect in DSS-exposed mice by activating the opioid pathway. **(A)** Body weight change. **(B)** Macroscopic damage score. **(C)** Histological damage score. **(D)** MPO activity. **(E)** Colon TNF-α level. **(F)** Colon IL-1β level. **(G)** Colon IL-6 level. **(H)** Colon IL-10 level. **(I)** Western blot analysis of iNOS, COX-2, TLR-4, and NF-κB expression in the colon. **(J)** Quantitative analysis of the relative contents of iNOS and COX-2. **(K)** Quantitative analysis of the relative contents of TLR-4 and NF-κB. The data are expressed as mean ± S.E.M. (*n* = 3–8). ^*^ *p* < 0.01, ^**^ *p* < 0.01, ^***^ *p* < 0.005, ^****^ *p* < 0.0001 *vs.* control group, ^##^ *p* < 0.01, ^###^ *p* < 0.005, ^####^ *p* < 0.0001 *vs.* DSS group, and ^$^ *p* < 0.01, ^$$^ *p* < 0.01 *vs.* HO + DSS group, one-way ANOVA with Tukey’s multiple comparisons test. Bars with different letters represent significant differences between groups by Fish’s LSD test (*p* < 0.05).

To examine the mechanisms of the protective effect of HO on DSS-induced colitis in mice, opioid receptor antagonist NH was used in the study. As shown in Figures 4A–D, HO treatment at a dose of 40 μg/kg significantly attenuated DSS-induced colitis in mice, as demonstrated by reducing body weight change, macroscopic scores (*p* < 0.0001), and histological scores (*p* = 0.0001), as well as colonic MPO activity (*p* = 0.0045), which were inhibited by pretreatment of NH. NH also countered anti-inflammatory effects of HO by significantly increasing colonic mucosal inflammatory cytokines TNF-α (*p* = 0.0149), IL-6 (*p* = 0.0085), and IL-1β (*p* = 0.0325), and decreasing anti-inflammatory cytokine IL-10 (*p* = 0.0063) in NH + HO + DSS group mice compared to the HO + DSS group (Figures 4E–H). In addition, HO obviously attenuated the DSS-induced increase in the expression of pro-inflammatory enzymes iNOS (*p* = 0.0104) and COX-2 (*p* < 0.0001) in the colon, which were completely inhibited by NH administration (Figures 4I,J). This suggests that HO shows anti-inflammatory effect through the opioid pathway. Moreover, to investigate the activation of inflammation involved in the DSS-induced colitis model, the NF-κB signaling pathway was detected. Western blotting results showed a significant increase in the protein expression levels of NF-κB p65 and TLR-4 in the colon tissue of DSS-exposed mice (*p* = 0.0016 and *p* = 0.0040) compared to the control group (Figures 4I,K). On contrary, treatment of HO significantly reduced the colonic protein levels of NF-κB p65 and TLR-4 in DSS-induced mice, and pretreatment with NH normalized the expression of NF-κB p65 (*p* = 0.0002) compared to HO + DSS group mice. Overall, these results indicate that HO prevents DSS-induced colitis in mice through inhibition of the NF-κB-mediated pro-inflammatory signaling pathway by activating the opioid pathway.

### 3.4 Protective effect of human opiorphin on intestinal barrier function and motility in dextran sodium sulfate-treated mice

**FIGURE 5:**
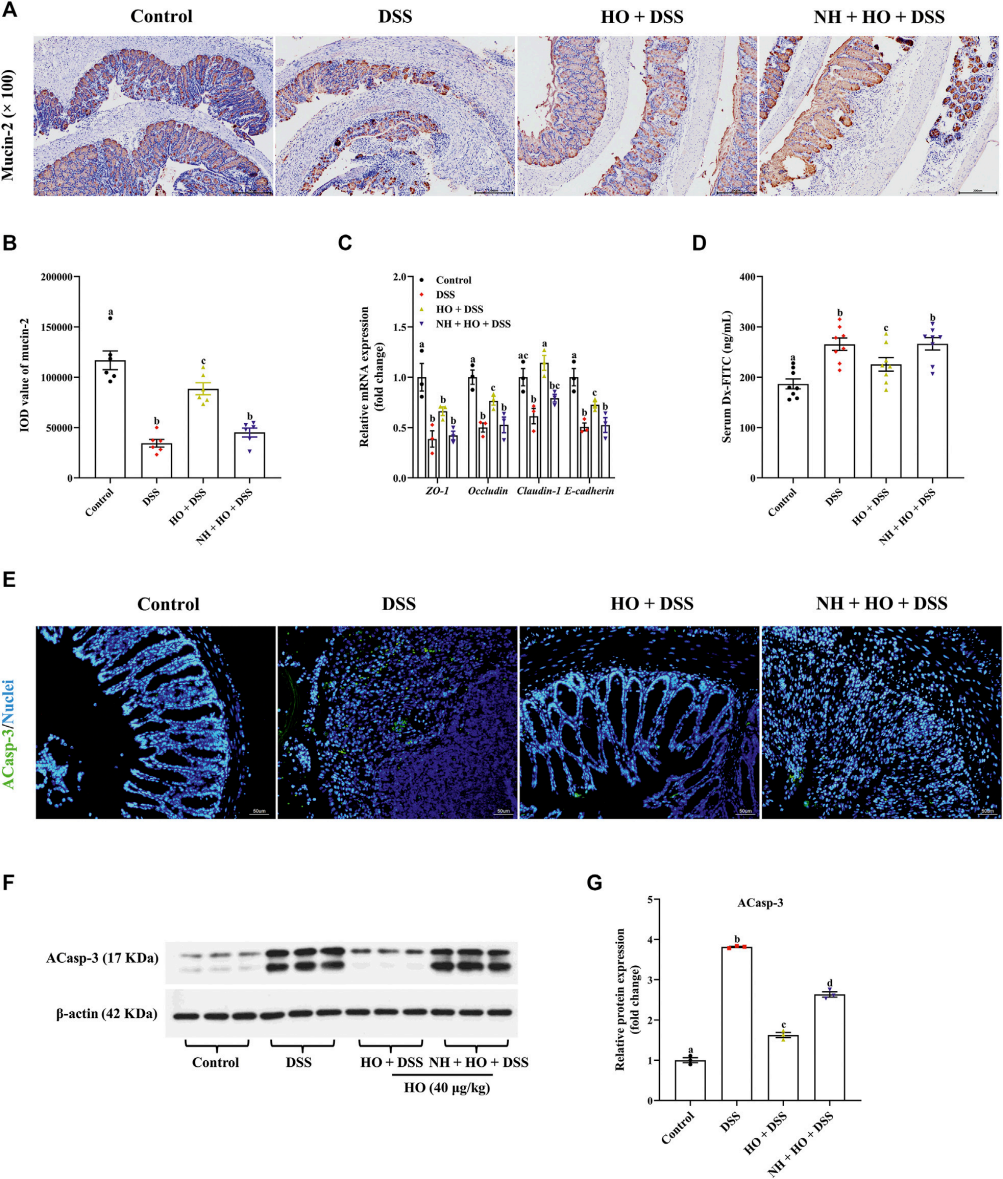
HO treatment improves intestinal barrier function in DSS-exposed mice through the opioid pathway. **(A)** Immunohistochemical staining with mucin-2 and E-cadherin in colonic sections. **(B)** Quantitative integrated optical density (IOD) analysis of colonic mucin-2. **(C)** Relative gene expression of tight junction proteins in the colon. **(D)** Serum Dx-FITC level. The data are expressed as mean ± S.E.M. (*n* = 3–8). **(E)** Apoptosis in the colonic mucosa of each group of mice. Green indicates the presence of ACasp-3, and Nuclei are blue. **(F)** Western blot analysis of ACasp-3 expression in the colon. **(G)** Quantitative analysis of the relative contents of ACasp-3. Bars with different letters represent significant differences between groups by Fish’s LSD test (*p* < 0.05).

To further investigate potential mechanisms of how HO attenuated DSS-induced colitis, we evaluated the effect of HO treatment on intestinal barrier function. Compared to the control group, immunohistochemistry analysis revealed that DSS exposure significantly decreased the expressions of mucin-2 (*p* < 0.0001) in the colon in DSS group mice, which were countered after HO administration (Figures 5A,B). Pretreatment with NH decreased the levels of mucin-2 (*p* < 0.0001) in the NH + HO + DSS group mice in comparison to the HO + DSS group. Moreover, AB-PAS staining of colonic segments also evidenced that DSS decreased goblet cell containing mucus and HO reversed it (Supplementary Figure S1). In addition, tight junction and adherent junction proteins of the intestine play crucial roles in maintaining the integrity of the intestinal epithelial barrier and inflammation. HO significantly increased the relative mRNA expressions of *occludin*, *claudin-1*, and *E-cadherin* in the colon of DSS-exposed mice (*p* = 0.0158, *p* = 0.0007, and *p* = 0.0357, respectively), all of which were remarkably reduced by pretreatment of NH (*p* = 0.0284, *p* = 0.0081, and *p* = 0.0489, respectively), suggesting that opioid receptors may be involved in the protective effects of HO on the intestinal barrier function (Figure 5C). Moreover, apoptosis analysis and Western blotting results found that DSS exposure increased the expression of ACaps-3 in colonic tissues (Figures 5E–G). It indicates that DSS could induce apoptosis of colonic epithelial cells and enhance leaky epithelium (Castro-Martinez et al., 2021). However, HO treatment significantly decreased ACaps-3 levels compared to the DSS group (*p* < 0.0001), and was obviously inhibited by NH administration (*p* < 0.0001). Furthermore, intestinal permeability was examined by measuring serum Dx-FITC levels. Compared to control group mice, DSS significantly elevated serum Dx-FITC levels (265.48 ± 12.34 versus 186.66 ± 9.42 ng/ml, *p* < 0.0001), which was reversed by HO administration (*p* = 0.0266) (Figure 5D), suggesting that HO decreased DSS-induced intestinal hyper-permeability. In contrast, NH pretreatment recovered intestinal permeability to that of DSS group mice. These results show that HO improved intestinal barrier function by enhancing the expression of tight junction proteins and restraining apoptosis of the epithelial cell. In addition, HO promoted the intestinal motility of DSS-induced colitis in mice by obviously decreasing total transit time (*p* = 0.0026, Supplementary Figure S2A) and increasing the defecation rate (without significant difference, Supplementary Figure S2B), which was reversed by NH pretreatment. These results demonstrate that HO prevents DSS-induced intestinal motility disturbances *via* activating opioid receptors. Taken together, our findings indicate that HO alleviates DSS-induced colitis in mice by maintaining gastrointestinal homeostasis through the opioid signaling pathway.

### 3.5 Effect of human opiorphin on endogenous enkephalins and the expression of mu opoid receptor and delta opoid receptor in dextran sulfate sodium-treated mice

To further investigate whether opioid receptors participate in the protective effect of HO, we first detected endogenous opioid peptide enkephalins in mice of each group.

**FIGURE 6:**
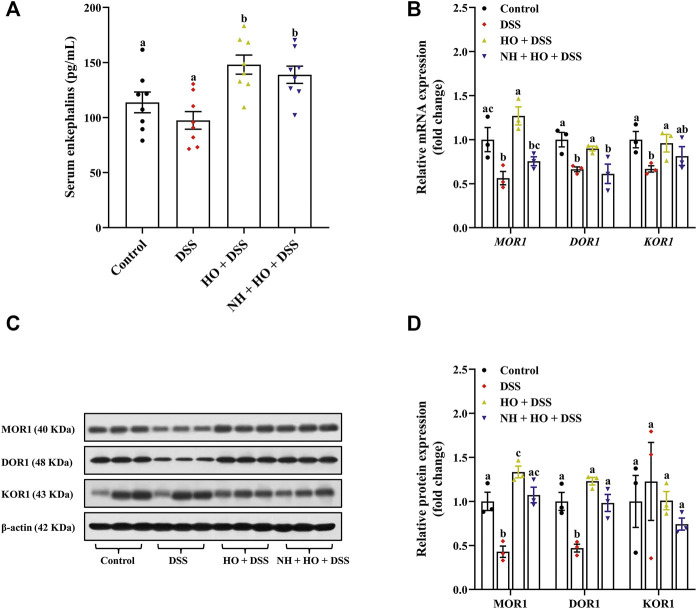
Effects of HO treatment on serum enkephalins concentration and expression of opioid receptors in DSS-exposed mice. **(A)** Serum enkephalins level. **(B)** Relative gene expression of opioid receptors in the colon. **(C)** Western blot analysis of opioid receptors expression in the colon. **(D)** Quantitative analysis of the relative contents of opioid receptors. The data are expressed as mean ± S.E.M. (*n* = 3–8). Bars with different letters represent significant differences between groups by Fish’s LSD test (*p* **<** 0.05).

As shown in Figure 6A, DSS exposure did not influence serum enkephalins concentration compared to control group mice. However, treatment with HO significantly increased serum enkephalins of DSS-treated mice compared to that of the control group and DSS group (*p* = 0.0081 and *p* = 0.0002, respectively). It is to be noted that NH did not alter the HO-induced increase of enkephalins in serum of DSS-exposed mice compared to the HO + DSS group, suggesting that HO inhibits the degradation of endogenous enkephalins. In addition, HO significantly enhanced the gene and protein expression of MOR1 (*p* = 0.0009 and *p* < 0.0001, respectively) and DOR1 (*p* = 0.0477 and *p* = 0.0001, respectively) in the colon of DSS-exposed mice (Figures 6B–D). Pretreatment with NH had no significant effect on protein levels of MOR1, DOR1, and KOR1 in NH + HO + DSS group mice compared to the HO + DSS group, but significantly decreased gene expressions of *MOR1* (*p* = 0.0056) and DOR1 (*p* = 0.0216). Taken together, these results indicate that HO inhibits the degradation of endogenous enkephalin and promotes the expression of MOR and DOR in DSS-treated mice.

### 3.6 Effects of human opiorphin on the expression of neutral endopeptidase and aminopeptidase N in dextran sodium sulfate-treated mice

**FIGURE 7:**
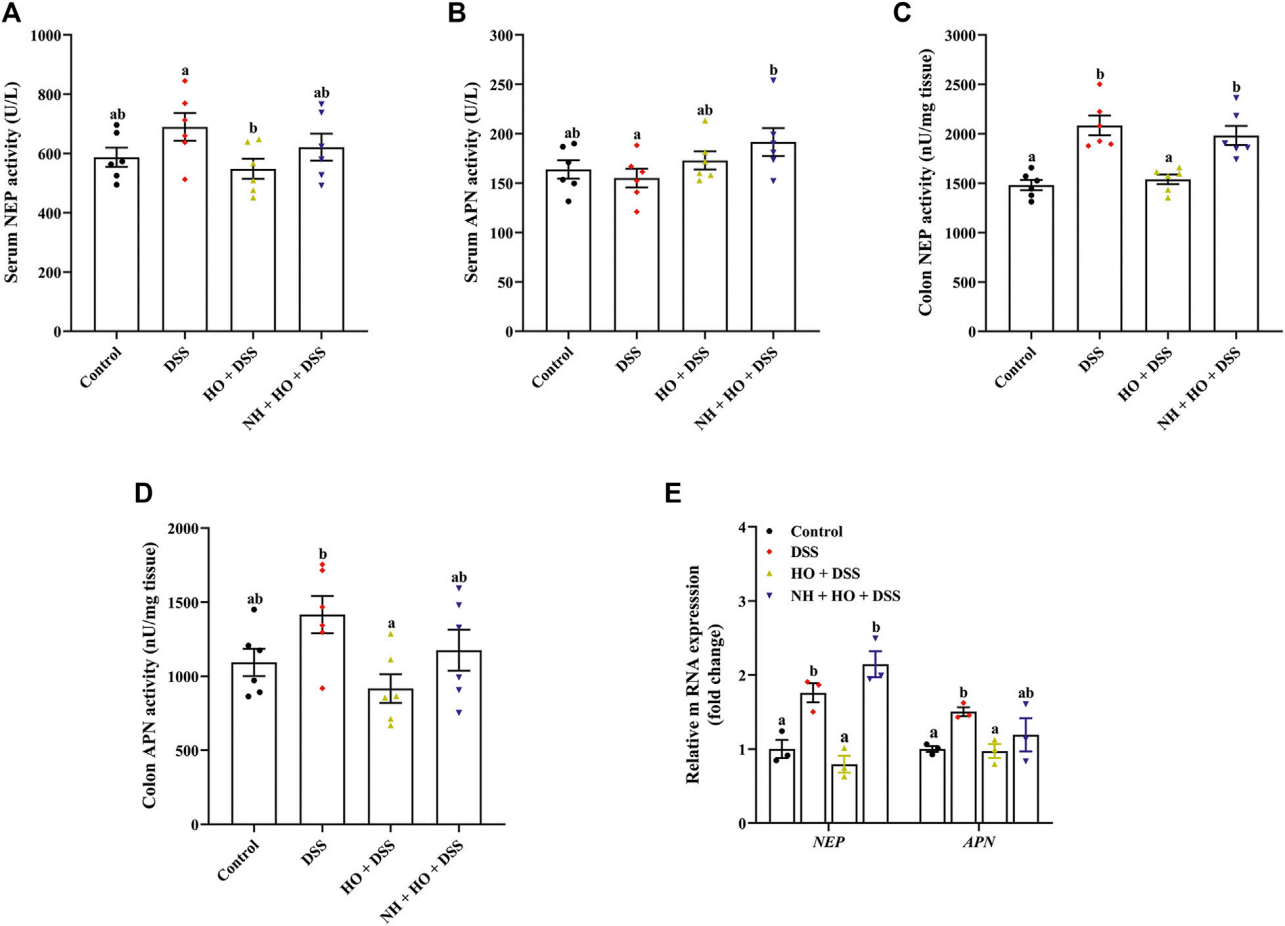
Effects of HO treatment on expression of NEP and APN in the serum and colon in DSS-exposed mice. **(A)** Serum NEP activity. **(B)** Serum APN activity. **(C)** Colon NEP activity. **(D)** Colon APN activity. **(E)** Relative gene expression of NEP and APN in the colon. The data are expressed as mean ± S.E.M. (*n* = 3–6). Bars with different letters represent significant differences between groups by Fish’s LSD test (*p* < 0.05).

To evaluate the relationship between endogenous enkephalins and the effects of HO on DSS-induced colitis, enkephalinases NEP and APN were also examined. Our results revealed that DSS exposure did not affect serum NEP and APN activities as well as the colonic APN activity (Figures 7A,B,D), while the NEP activity in the colonic tissue was significantly increased in DSS group mice compared to the control group (2084.62 ± 99.66 *versus* 1481.41 ± 52.17 nU/mg, *p* < 0.0001) (Figure 7C). On the contrary, HO treatment remarkably decreased the NEP activity both in serum and colonic tissue in DSS-exposed mice (*p* = 0.0221 and *p* < 0.0001), of which the NEP activity in colonic tissue was normalized by NH (*p* = 0.0007). In accordance with this, we also found that the gene expressions of NEP and APN in the colon were markedly elevated in the DSS-exposed mice with colitis (*p* = 0.0044, *p* = 0.0230), which were absolutely countered after HO treatment (Figure 7E). Pretreatment with NH recovered the reduction of these gene expressions induced by HO to the levels of DSS group mice. Therefore, these data indicate that HO attenuates the expression of NEP and APN in mice with DSS-induced colitis.

## 4 Discussion

The available evidence has demonstrated that NEP/APN inhibitors are capable of reducing the colonic inflammatory response. Systemic administration of sialorphin attenuates TNBS-induced colitis in mice by activating MOR and KOR (Sałaga et al., 2017). However, more recent study on natural enkephalinase inhibitors suggests that sialorhin has no effect on DSS-induced colitis, and human opiorphin does not alleviate colitis induced by TNBS in mice (Sałaga et al., 2017; Sobocińska et al., 2020), the cause of which still remains unclear and needs further investigations. In the present study, our results establish that central administration of HO effectively prevents DSS-induced colitis in mice. Notably, we confirm that HO treatment restrains the activity and expression of NEP and APN in the colon, and exhibits anti-inflammatory effect mediated by MOR and DOR through the protection of endogenous enkephalins. Moreover, we also found that HO improves intestinal barrier function by enhancing the expression of tight junction proteins in DSS-exposed mice.

Opioid peptides, acting as an important cross talk between the neuroendocrine and immune systems, play a significant clinical role in immune-mediated diseases, particularly in regulating neuroimmune, vascular immune, and cellular immune, as well as auto-immunoregulation (Owczarek et al., 2011; Sałaga et al., 2013). However, these peptides are quickly degraded by peptidases NEP or APN, which severely limit the anti-inflammatory effect. The endogenous opioid system is considered one of the most attractive pharmacological targets for protease-based anti-inflammatory therapies. Therefore, inhibition of enzymatic degradation and an increase of endogenous opioid peptide levels will be one of the possible approaches to prolong their activity and attenuate inflammatory response in IBD. NEP inhibitors, thiorphan and acetorphan, are identified to exert anti-diarrheal activity in rodents *via* the protection of endogenous enkephalins (Marҫais-Collado et al., 1987; Sałaga et al., 2013). Recent studies show that a natural inhibitor of NEP/APN, sialorphin, attenuates TNBS-induced colitis in mice (Sałaga et al., 2017; Sobocińska et al., 2020). Similarly, our results revealed that i.c.v. injection of HO reduced clinical symptoms associated with ulcerative colitis in DSS-exposed mice, including body weight change, fecal blood, diarrhea, colon shortening, and DAI scores, as well as histological damage scores, MPO, and inflammatory biomarkers. Moreover, the effect of HO showed superior ability to that of the positive drug 5-ASA. These data indicate that HO effectively attenuates DSS-induced colitis in mice.

DSS exposure activates lymphocytes and M1-type macrophages, resulting in inhibition of cell proliferation and activation of the Th1/Th2 immune response (Strober, et al., 2002). DSS-induced colitis in a mouse model is popularly used for mimicking human IBD and is characterized by the production and secretion of inflammatory cytokines from immune cells (Kiesler et al., 2015). It is well known that DSS upregulates the secretion of IFN-γ, TNF-α, IL-4, IL-5, IL-6, and IL-17 mediated by T-cell activation, which are the main drivers of IBD (Kiesler et al., 2015; Imam et al., 2018). Increasing studies have demonstrated that targeting Th1, Th2, Th17, and Treg cells are effective in the treatment of colitis (Brand, 2009; Boehm et al., 2012). Th2-derived cytokine IL-10 in human beings and animals has been reported to be highly associated with various anti-inflammatory effects. In accordance with our study, Th1-derived TNF-α, Th17-derived IL-1β, and IL-6 were significantly increased in DSS-induced colitis mice compared to the control. Treatment with HO subsequently decreased systemic and colonic pro-inflammatory cytokine (TNF-α, IL-1β, and IL-6) levels, while it increased anti-inflammatory cytokine IL-10 in DSS-treated mice. Notably, HO at a dose of 40 μg/kg remarkably suppressed mRNA expression levels of TNF-α, IL-1β, and IL-6, and upregulated the IL-10 expression in the colon in DSS group mice, or even more than that in the HO (20 μg/kg) + DSS group. These data indicate that HO treatment with a 40 μg/kg dose has profound anti-inflammatory properties. It is well known that IL-10 is a key immunoregulatory cytokine secreted by immune and nonimmune cells, which plays a central role in regulating intestinal inflammation in humans and mice through activation of the IL-10 receptors and the epidermal growth factor receptor in macrophages (Lu et al., 2014; Dayagi et al., 2021). Several research works supported that IL-10 is highly relevant to IBD, and IL-10−/− mice would spontaneously develop colitis, which may be related to the production of IL-22 (Gunasekera et al., 2020). In general, IL-10 levels are not changed or even increased in patients with ulcerative colitis and the mice model of DSS-induced colitis through regulating the innate and adaptive immune response. However, with the decline in the ability of immunomodulation, IL-10 levels would be decreased. Some studies reported that the expression of IL-10 in the colon was reduced in mice with DSS-induced colitis, and Wogonin enhanced IL-10 production by inducing transcript factor HIF-1α expression *via* the AKT/GSK3β signal pathway (Wu et al., 2021). Therefore, in the present study, HO might increase the IL-10 expression and display anti-inflammatory activities in the intestinal tissue. However, the mechanism needs to be further investigated. Moreover, HO significantly decreased the protein levels of pro-inflammatory enzymes iNOS and COX-2 in DSS-exposed mice. iNOS and COX-2 are proven to be induced at sites of inflammation in response to pro-inflammatory cytokines, such as TNF-α and IL-1β (Wang and Dubois, 2010). Clinical studies have identified that iNOS and COX-2 promote the expression of each other in inflammation and play important roles in the development of colitis lesions (Kim et al., 2005; Wang et al., 2015). Therefore, HO exhibits anti-inflammatory properties and attenuates DSS-induced colitis through its immunomodulatory effects.

To evaluate underlying mechanisms by which HO exhibits protective effect on DSS-induced colitis in mice, protein levels of TLR-4 and NF-κB in the colon were examined. It is well known that the TLR/NF-κB pathway is considered one of the key regulators in the pathogenesis of IBD (Kordjazy et al., 2018; Lu et al., 2018). TLRs lead to the secretion of pro-inflammatory mediators and thus induce inflammatory response (Cen et al., 2018). Previous studies have demonstrated that the TLR expression in the colon is upregulated in an animal model of colitis induced by DSS (Majumder et al., 2017). In addition, the production of pro-inflammatory cytokines is blocked and symptoms of colitis are ameliorated in TLR4-deficient mice (Hwang et al., 2018). NF-κB, acting as a key mediator of the inflammatory response, induces the expression of pro-inflammatory cytokines, such as COX-2, TNF-α, IL-1β, and IL-6 (Algieri et al., 2014; Song et al., 2019). In our study, DSS increased the protein levels of TLR-4 and NF-κB p65 in the colon, suggesting that DSS activates the TLR-4/NF-κB pathway and results in a pro-inflammatory response in DSS-induced colitis mice. This is in line with previous studies that the TLR-4 expression and NF-κB activation are greatly upregulated in the colonic tissues of patients with IBD and mice with DSS-induced colitis (Cario and Podolsky, 2001; Wang et al., 2020). More importantly, our results confirmed that HO treatment normalized the protein expression levels of TLR-4 and NF-κB. These results indicate that HO alleviates DSS-induced colitis probably through suppressing the TLR-4/NF-κB pathway-mediated pro-inflammatory response. However, pretreatment with NH normalized the expression of NF-κB p65 but did not reverse the TLR4 expression. It has been confirmed that opioids have high efficacy activity at innate immune pattern recognition binding sites but do not bind to TLR4 (Thomas et al., 2022). Therefore, TLR4 may not be the only TLR contributing to the pro-inflammatory response and the behavioral outcomes of opioids. A previous study reported that opioid receptor agonist, BU08070, produced concentration-dependent inhibition of LPS-induced NF-κB activation, and exerted anti-inflammatory effect in mice with experimental colitis through activation of mu and delta opioid receptors (Zielińska et al., 2015). This suggests that the anti-inflammatory properties of HO through activating opioid receptors are associated with NF-κB engagement. The possible involvement of other signaling pathways remains unclear and needs to be further studied.

The intestinal epithelium cells are critical for regulating the intestinal permeability and the mucosal barrier integrity, and thus for preventing inflammation and colonic damage (Van Der Gracht et al., 2016). Pro-inflammatory cytokines have been proven to induce intestinal epithelial barrier dysfunction, which further augments the pathophysiological state of IBD (McGuckin et al., 2008; Michielan and D’Incà, 2015). Previous studies have demonstrated the DSS-induced pro-inflammatory response is often accompanied by intestinal barrier dysfunction with decreased tight junction proteins and mucin depletion (Kim et al., 2018; Kim et al., 2019). In addition, mucin-2 knockout mice are reported to be more susceptible to DSS-induced colitis and mucin-2 depletion is generally considered a characteristic of UC patients (Dorofeyev et al., 2013). In our study, HO treatment protected against the depletion of mucin-2 and goblet cells in the colon of DSS-induced colitis mice. Moreover, the intestinal barrier integrity is mainly maintained and modulated by tight junction proteins, the reduction of which leads to intestinal barrier disruption in mice with DSS-induced colitis (Eichele and Kharbanda, 2017). Consistent with previous studies, we observed a significant decrease in the mRNA expression of *ZO-1*, *occludin*, and *claudin-1*, and *E-cadherin* in the colon of DSS-treated mice, which were normalized after treatment with HO. These findings indicate that HO enhanced the expression of tight junction proteins and improved intestinal barrier integrity in mice with DSS-induced colitis. In addition, HO treatment decreased ACaps-3 levels, suggesting that HO inhibits DSS-induced apoptosis of colonic epithelial cells and leaky epithelium. Intestinal barrier dysfunction goes hand in hand with increased intestinal permeability in patients with IBD and animal models of DSS-induced colitis (Gerova et al., 2011). Here, serum Dx-FITC levels revealed that HO obviously decreased DSS-induced intestinal hyper-permeability. Furthermore, we also demonstrated that HO improved the intestinal motility of DSS-induced colitis in mice by decreasing total transit time. It has been reported that DSS exposure delays gastrointestinal emptying, disturbs intestinal motility, and leads to constipation (Rtibi et al., 2018). Constipation is a functional gastrointestinal disorder characterized by slow transit or reduction in the frequency of bowel movements (Bai et al., 2016), and it is recognized as a characteristic of DSS-induced colitis (Wadie et al., 2012). Our previous study has verified that HO induces colonic contractions (Tian et al., 2009). The increase in colonic contraction may help relieve constipation. Therefore, this indicates that HO prevents DSS-induced intestinal motility disturbances and attenuates constipation in DSS-induced colitis in mice. Taken together, these data suggest that HO maintains intestinal immune homeostasis, improves intestinal barrier function, and enhances colonic transit. Accordingly, our results confirmed for the first time that HO ameliorates DSS-induced colitis *via* inhibiting TLR4/NF-κB pathway activation and regulating intestinal homeostasis.

To further explore potential mechanisms of how HO treatment prevented DSS-induced colitis in mice, serum enkephalins and colonic opioid receptors as well as enkephalinases NEP and APN were investigated. It has been reported that opioids affect a series of physiological processes, including nociceptive transmission, respiration, feeding, and mood-related behavior, as well as gastrointestinal motility, and immune and inflammatory response. Multiple studies have confirmed that opioids and opioid-like peptides exert pronounced anti-inflammatory efficacy and are effective in the prevention and therapy of colitis in mice through activation of opioid receptors in the intestine. For example, selective MOR agonists, DALDA and DAMGO, are shown to improve TNBS-induced colitis, and MOR and DOR knockout mice are found more susceptible to colitis than the wild type (Philippe et al., 2003; Reiss et al., 2016). Endogenous opioids, such as enkephalins, endomorphins, and dynorphin, are also proven to have an anti-inflammatory and antitumor response (Owczarek et al., 2011; Wilenska et al., 2018; Tuo et al., 2020). Numerous investigations indicate enkephalins and other opioids participate in regulating intestinal motility (De Schepper et al., 2004; Wood and Galligan 2004). It suggests that the endogenous opioid system plays a critical role in maintaining gastrointestinal immune homeostasis. Consistent with previous studies on the protective effect of HO on enkephalins from enzymatic degradation, in our study, HO supplementation induced a significant increase in serum enkephalin and obviously increased gene and protein expressions of MOR1 and DOR1 in the colon tissues of DSS-treated mice. Moreover, opioid receptor antagonist naloxone absolutely inhibited the protective effects of HO on DSS-induced colitis, including clinical symptoms, colonic inflammation, and intestinal barrier integrity. These data substantiate that HO alleviates DSS-induced colitis through activating the enkephalins-dependent mu and delta opioid pathway. NEP and APN, the major enkephalinases, have been reported to regulate opioid signaling in the gut, which is considered the cause of impaired intestinal motility, intestinal barrier dysfunction, and colonic inflammation in IBD patients (Sałaga et al., 2017). NEP and APN recruit immune cells and potentiate cytokines’ and chemokines’ pro-inflammatory properties, and thus lead to IBD (Vergnolle, 2016). DPP IV or APN inhibitors are reported to increase transcription, synthesis, and secretion of TGFβ1, and suppress the production of pro-inflammatory cytokines through inhibition of enkephalin degradation (Bank et al., 2006), suggesting that peptidase inhibitors would play beneficial effect on colonic inflammation *via* an opioid pathway. In addition, previous studies have proven that NEP and APN are over-expressed in IBD patients (Sałaga et al., 2017). Similarly, our results revealed that the activity and gene expression of NEP and APN in the colon were significantly increased in mice with DSS-induced colitis, which were normalized after HO treatment. Overall, our data demonstrated that HO attenuates DSS-induced colitis by blocking NEP and APN through activation of the endogenous enkephalin-dependent opioid pathway. However, a recent study reports that systemic administration of human opiorphin has no effect on TNBS-induced colitis in mice (Sobocińska et al., 2020). Although inflammatory pathways induced by DSS and TNBS in experimental models of colitis in mice are similar, DSS-induced ulcerative colitis is more severe than TNBS-induced colitis. This suggests that central administration of HO shows more effectiveness than a systemic injection, which may be due to the high abundant opioid receptors in the central nervous system. Therefore, more extensive studies are needed to evaluate the protective effect of HO on IBD, especially the clinical potential in human subjects.

## 5 Conclusion

In summary, our results indicated that central administration of HO exhibited anti-inflammatory effect and effectively alleviated DSS-induced colitis in mice by inhibiting TLR-4/NF-κB pathway-mediated pro-inflammatory response and improving intestinal barrier function. Notably, we confirmed that the protective effect of HO might be associated with enkephalin-dependent mu and delta opioid pathways through the blockage of NEP and APN activation. Therefore, the opioid system plays a critical role in maintaining gastrointestinal homeostasis, and targeting the endogenous opioid system with peptidase inhibitors intervention may be a promising therapeutic strategy for the prevention or treatment of IBD. Our findings will provide a theoretical basis for future clinical research in the treatment of IBD.

## Data Availability

The original contributions presented in the study are included in the article/Supplementary Material; further inquiries can be directed to the corresponding authors.
