# Supplementary material for: Central administration of human opiorphin alleviates dextran sodium sulfate-induced colitis in mice through activation of the endogenous opioid system
Source: Front Pharmacol. 2022 Sep 13;13:904926. doi: 10.3389/fphar.2022.904926 (PMC9513434; doi:10.3389/fphar.2022.904926)
Supplement: Supplementary file 1 [file Table1.DOC]

**Supplementary material**

**Materials and Methods**

### *Histopathological analysis*

The colonic tissues were fixed with 4% paraformaldehyde for 12 h, and then, processed to prepare 4 µm paraffin sections for periodic acid-schiff (PAS) staining (Beijing Solarbio Science & Technology, Beijing, China). The slides were then studied by light microscope for observation and photography.

***Intestinal motility assay***

According to previous study , carmine red was used to evaluate intestinal motility. Briefly, carmine red was dissolved in PBS. Each mouse was orally gavaged with 6% carmine red (200 µL). After that, fecal pellets of each mouse were monitored and counted at 10 min intervals until the production of the first red fecal pellet, and the interval period was recorded as the total transit time. Defecation rate was calculated by the number of fecal output relative to the total transit time.

**Results**

**
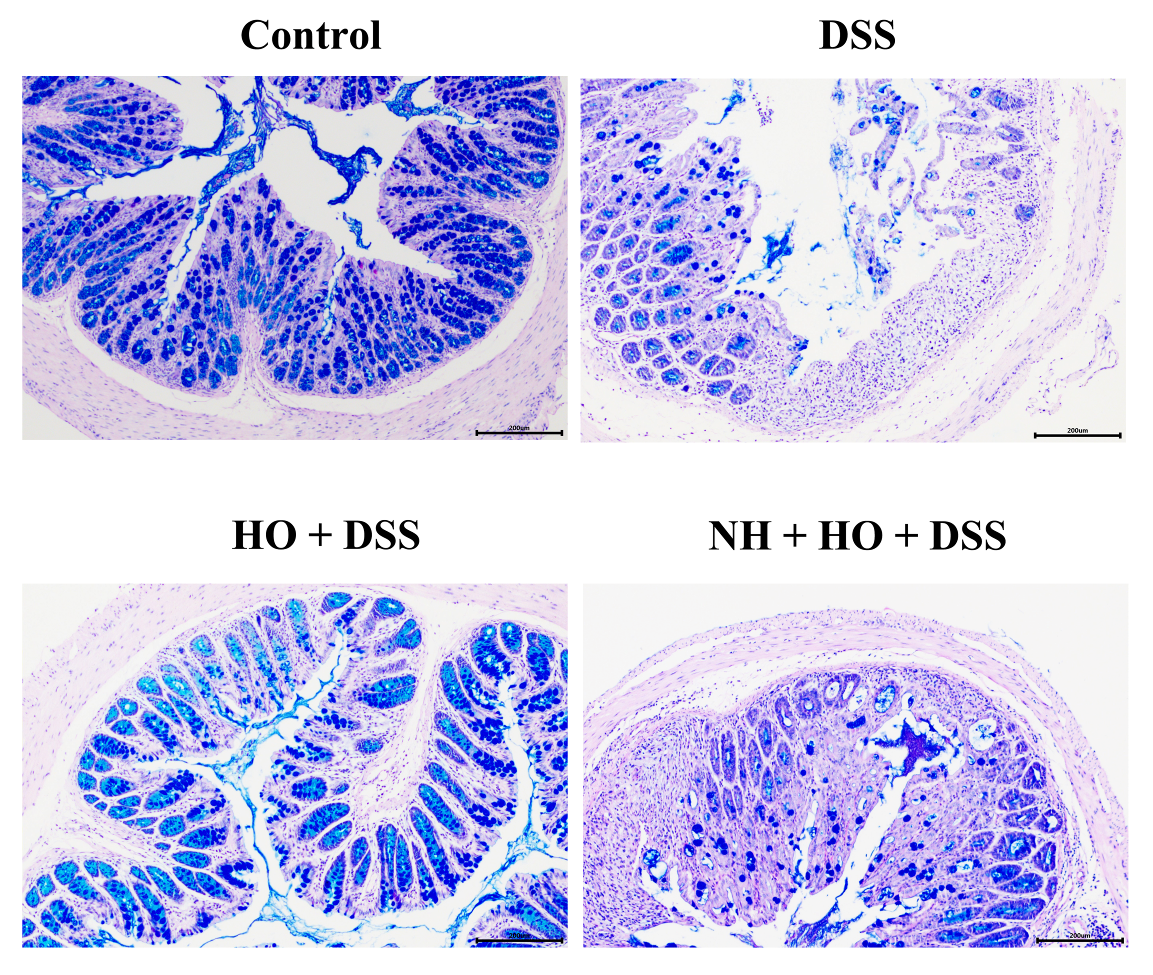
**

**Figure S1** AB-PAS staining of colonic segments in mice with each group. The decrease of mucopolysaccharides represents depletion of goblet cells.


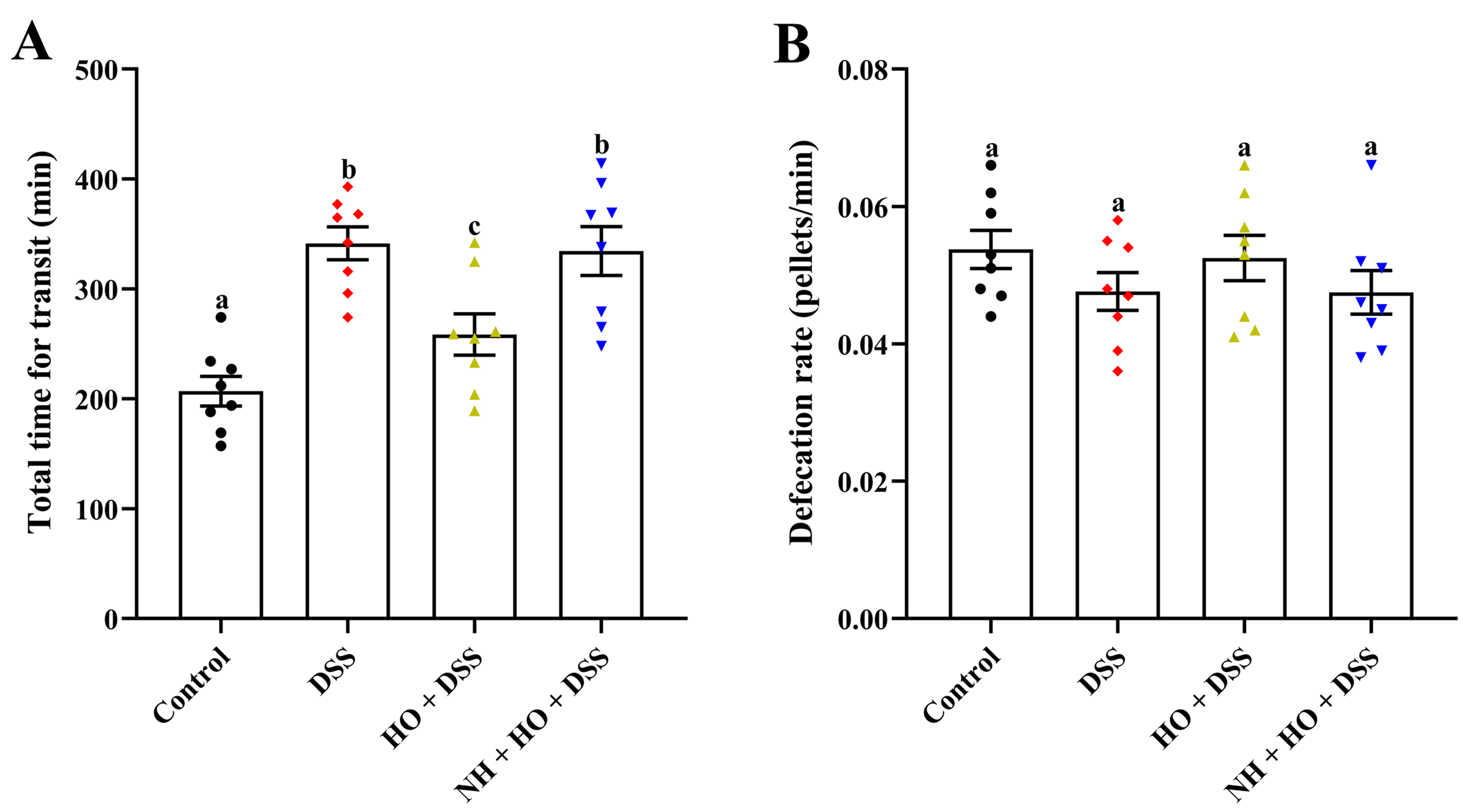


**Figure S2** Effect of HO on gut motility in DSS-exposed mice. **(A)** Transit time. **(B)** Defecation rate. The data are expressed as mean ± S.E.M. (n = 8). Bars with different letters represent significant differences between groups by Fish’s LSD test (*P* < 0.05).

****Table S1 The criteria for scoring mouse daily disease activity index (DAI)****

| Parameters | Score | Description |
| --- | --- | --- |
| Relative body weight change | 0 | increase or no change |
|  | 1 | Decreased by 1%-5% |
|  | 2 | Decreased by 5%-10% |
|  | 3 | Decreased by 10%-15% |
|  | 4 | Decreased more than 20% |
| Stool consistency | 0 | Normal |
|  | 1 | Loose stool |
|  | 2 | Mucoid stool |
|  | 3 | Diarrhoea |
| Fecal blood | 0 | No blood |
|  | 1 | Positive benzidine probe (blue) |
|  | 2 | Visual pellet bleeding or haemorrhage |

**Table S2 The criteria for** scoring macroscopic colonic damage

| Parameters | Score | Description |
| --- | --- | --- |
| Ulcer score | 0 | Dots of ulceration: none |
|  | 1 | Dots of ulceration: 1 |
|  | 2 | Dots of ulceration: 2 |
|  | 3 | Dots of ulceration: ≥ 3 |
| Colon shortening | 0 | Less than 15% |
|  | 1 | 15%-25% |
|  | 2 | More than 25% |
| Diarrhoea | 0 | Absence |
|  | 1 | Presence |
| Fecal blood | 0 | Absence |
|  | 1 | Presence |
| Haemorrhage | 0 | Absence |
|  | 1 | Presence |
| Colon thickness | *n* | Colon thickness of *n* mm represents *n* scoring points |

**Table S3 The criteria for** scoring histological colonic damage

| Parameters | Score | Description |
| --- | --- | --- |
| Goblet cell depletion | 0 | Absence |
|  | 1 | Presence |
| Crypt abscesses | 0 | Absence |
|  | 1 | Presence |
| Extent of muscle thickening | 0 | Normal |
|  | 1 | Mild |
|  | 2 | Moderate |
|  | 3 | Extensive |
| Destruction of the mucosal layer | 0 | Normal |
|  | 1 | Mild |
|  | 2 | Moderate |
|  | 3 | Extensive |
| Cellular infiltration | 0 | No infiltrate |
|  | 1 | Infiltrate around crypt basis |
|  | 2 | Infiltrate in lamina muscularis mucosa |
|  | 3 | Extensive infiltrate in mucculatis mucosa |

**Table S4** The macroscopic colonic damage score in each group mice

| Groups | Ulcer score | Colon shortening | Diarrhoea | Fecal blood | Haemorrhage | Colon thickness |
| --- | --- | --- | --- | --- | --- | --- |
| Control | 0.250 ± 0.164^a^ | 0.250 ± 0.164^a^ | 0.125 ± 0.125^a^ | 0.000 ± 0.000^a^ | 0.000 ± 0.000^a^ | 0.721 ± 0.044^a^ |
| DSS | 1.625 ± 0.263^bd^ | 1.625 ± 0.263^bc^ | 1.000 ± 0.000^b^ | 1.000 ± 0.000^b^ | 0.750 ± 0.164^b^ | 0.996 ± 0.047^bd^ |
| HO (5 μg/kg) + DSS | 2.000 ± 0.267^b^ | 1.750 ± 0.164^b^ | 0.750 ± 0.164^bc^ | 0.875 ± 0.125^bd^ | 0.625 ± 0.183^bc^ | 1.075 ± 0.088^b^ |
| HO (10 μg/kg) + DSS | 1.250 ± 0.313^cd^ | 1.000 ± 0.327^cd^ | 0.750 ± 0.164^bc^ | 0.625 ± 0.183^bc^ | 0.500 ± 0.189^b^ | 0.859 ± 0.043^cd^ |
| HO (20 μg/kg) + DSS | 0.375 ± 0.183^a^ | 0.375 ± 0.183^ad^ | 0.375 ± 0.183^ac^ | 0.500 ± 0.189^cd^ | 0.375 ± 0.183^ab^ | 0.698 ± 0.059^ac^ |
| HO (40 μg/kg) + DSS | 0.625 ± 0.263^ac^ | 0.500 ± 0.267^ad^ | 0.500 ± 0.189^ac^ | 0.250 ± 0.164^ac^ | 0.125 ± 0.125^a^ | 0.718 ± 0.050^ac^ |
| 5-ASA + DSS | 0.625 ± 0.263^ac^ | 0.625 ± 0.263^ad^ | 0.500 ± 0.189^ac^ | 0.375 ± 0.183^ac^ | 0.250 ± 0.164^ac^ | 0.656 ± 0.062^ac^ |

Macroscopic scoring of colonic damage is assessed based on the criteria for different damage parameters. Values are expressed as mean ± S.E.M. (n = 8). Different letters in the same column represent significant differences between groups by Fish’s LSD test (*P* < 0.05). HO, human opiorphin; DSS, dextran sulphate sodium.

**References**

Yano, J.M., Yu, K., Donaldson, G.P., Shastri, G.G., Ann, P., Ma, L., et al. (2015). Indigenous bacteria from the gut microbiota regulate host serotonin biosynthesis. Cell, 161, 264-276. doi:10.1016/j.cell.2015.02.047
